# Improved YOLOv8-SST for accurate detection of small floating objects in complex water environments

**DOI:** 10.1371/journal.pone.0340822

**Published:** 2026-01-08

**Authors:** Huizhen Dong, Jianjun Li, Xi He, Mingchao Zhang

**Affiliations:** 1 College of Civil Engineering and Architecture, Zhejiang University of Water Resources and Electric Power, Hangzhou, China; 2 College of Mechanical and Electrical Engineering, China Jiliang University, Hangzhou, China; 3 Zhejiang School of Computer Science and Technology University of Water Resources and Electric Power, Hangzhou, China; Kafkas University: Kafkas Universitesi, TÜRKIYE

## Abstract

In response to the urgent need for water environment protection, this study proposes an improved algorithm for detecting floating objects on the surface of water: You Only Look Once version 8- Small Surface Targets (YOLOv8-SST). This algorithm aims to address the impacts of illumination variations and water surface distortion on floating object detection, as well as missed and false small object detections in complex aquatic scenarios. First, to mitigate the noise interference introduced during the downsampling process of the backbone network in complex aquatic environments, a C2fBF (C2f-BiFormer) module, based on the BiFormer dual-layer routing attention mechanism, was developed. This module effectively preserves fine-grained contextual feature information during feature extraction. Then, the conventional loss function was replaced with a more effective Inner-Complete Intersection over Union (Inner-CIoU) loss under auxiliary bounding boxes, allowing the model to adjust the size of auxiliary boxes more flexibly during detection and thereby improving detection accuracy. Finally, the adaptive moment estimation (Adam) optimizer in the original algorithm was replaced with the second-order clipped stochastic optimization (Sophia) optimizer to improve the generalizability of the model. On a combined dataset integrating FloW-Img, WSODD, and our self-collected data, YOLOv8-SST outperformed the baseline YOLOv8n, achieving a 3.1% increase in mean average precision (mAP)@0.5 and a 5.0% increase in mAP@0.5:0.95. These results demonstrate the effectiveness and robustness of the proposed method for small object detection in challenging natural water environments.

## Introduction

Currently, the increasing prevalence of waterborne debris in lakes, rivers, and urban water bodies is exacerbating the global water pollution crisis. The hazards posed by surface litter can be summarized as follows. (1) When submerged for extended periods, plastic waste releases harmful chemicals that degrade water quality. (2) Floating debris prevents light penetration, disrupting the existing ecological balance. (3) Plastic waste gradually breaks down into microplastics, which can be ingested by aquatic organisms and enter the human food chain, posing significant health risks. (4) Accumulated waste severely hinders vessel navigation. Consequently, effective surface debris management has garnered widespread attention worldwide [1]. Research indicates that China is one of the countries with the most extensive river networks in the world, covering a total area of 5.098 million square kilometers, which accounts for 53.1% of its national territory [2]. Therefore, managing the waste in inland water bodies has become a critical issue, making the protection of water resources an urgent need.

With the advancement of unmanned technologies and computer vision, traditional manual inspections of aquatic environments are increasingly being replaced by intelligent systems. Unmanned surface vehicles (USVs) are autonomous vessels that do not require human control and can navigate water surfaces according to predefined tasks via sensor technologies, demonstrating enhanced capabilities in terms of executing hazardous and time-consuming missions [3]. USVs are now widely applied in various fields, including port inspections, fishery monitoring, and maritime management, encompassing tasks such as object detection and environmental monitoring [4, 5]. The use of USVs to detect floating debris on water surfaces represents a highly effective approach. The evaluation of object detection technologies is crucial for maximizing the recognition performance of USVs, and deep learning-based object detection algorithms have gained significant attention because of their high object recognition accuracy.

In recent years, numerous detection models have emerged to enhance the ability to identify floating debris on water surfaces. Xiali Li et al. [6] aimed to improve the real-time detection performance of small floating objects on water surfaces by simplifying the detection scales of YOLOv3 from three to two. In addition, to ensure detection accuracy for small targets, the anchor boxes in the training dataset were re-clustered to replace some prior anchors of the original YOLOv3 that were not suitable for the dataset. Jiasheng Xu et al. [7] sought to enhance both the detection accuracy and computational efficiency of nearshore floating object detection under complex environments. Based on YOLOv3, they introduced an Efficient Channel Attention Network (ECANet) mechanism integrated with convolutional operators to prioritize key regions, thereby improving detection precision. Furthermore, the incorporation of the Spatial Pyramid Pooling (SPP) module facilitated the fusion of features from different receptive fields, enabling the model to effectively capture cross-scale information. Experimental results showed that the mean average precision (mAP) reached 93.73%, an increase of 6.87%, while the frame rate (FPS) reached 35.78, representing an improvement of 2.21 FPS over the original model. Zhang Lili et al. [8] proposed a single-stage object detection model, EYOLOv3, which employed a multi-scale feature extraction and fusion module to enhance the network’s feature representation capability. Additionally, a more effective clustering algorithm was used to analyze the size characteristics of floating objects to design anchor boxes, improving object detection efficiency. A focal loss function was also introduced to effectively address the problem of sample imbalance. Wei Tang et al. [9] addressed the challenges of detecting small floating objects and the high computational burden of existing detection algorithms, which hinder real-time operation on embedded devices. They proposed an improved YOLOv4-based detection method by first replacing the YOLOv4 backbone with MobileNetV3. Moreover, a negative feedback mechanism was introduced, using the proportion of small-object loss to the total loss during training as feedback for selective data augmentation, thereby enhancing detection accuracy for small targets. Experimental results demonstrated that, compared with the original YOLOv4, the proposed model reduced the number of parameters by 82.4% and increased detection speed by 52%.

Xiaohong Zhang et al. [10] proposed a hybrid attention mechanism that supports long-range interactions between channels while maintaining a direct correspondence between each channel and its weight. Based on this attention mechanism, an adaptive feature extraction module was developed to capture object features even in the presence of feature loss caused by downsampling. Additionally, a dedicated detection layer for small objects was introduced to enhance the detection performance of small floating objects.He Li et al. [11] presented a novel method based on an improved YOLOv5s. They designed a new data augmentation strategy for small objects and applied different coordinate attention pooling methods at various levels of the feature extraction network to enhance the effective feature representation of small targets, thereby improving detection accuracy. Compared with the original YOLOv5, the proposed method achieved a 15.7% increase in precision, reduced the false detection rate for small-object tasks by 83%, reached an edge test accuracy of 92.01%, and attained a frame rate of 33 FPS.Fuxun Chen [12] incorporated a shallow-layer small-object detection head into the YOLOv5 model, integrating spatial and semantic features to maximally preserve the critical characteristics of small floating objects. Moreover, the CIoU loss was replaced with SIoU, which considers the orientation between the ground truth and predicted boxes, thereby improving detection effectiveness for small water surface objects. The proposed improvements, through better integration of shallow and deep features, effectively addressed missed detections. Compared with the original YOLOv5, the enhanced model showed significant improvements in both average precision (AP) and recall (R), increasing by 5% and 6.1%, respectively.Xingshuai Yang et al. [13] proposed a novel detection model named YOLOv5_CBS. First, a non-compressed coordinate attention (CA) mechanism was incorporated into the C3 module to construct the C3-CA-Uncompress Bottleneck (CCUB) module, expanding the receptive field and enhancing focus on key features. Second, the original Path Aggregation Network (PAN) was replaced with a bidirectional feature pyramid network (BiFPN) to improve multi-scale feature fusion and information mining capabilities. Finally, the SCYLLA-IoU (SIoU) loss function was adopted to replace the CIoU loss, accelerating convergence and enhancing regression accuracy. Collectively, these modifications significantly strengthened the model’s feature extraction and detection performance.

Ning Li [14] proposed a detail-enhanced noise suppression YOLOv6 detection algorithm (DENS-YOLOv6) based on YOLOv6. First, to better capture detailed features of small objects, a detail information enhancement module (DIEM) based on atrous convolution was designed. Second, to suppress noise interference affecting small objects, an adaptive noise suppression module (ANSM) was developed. Finally, to improve training stability and convergence speed, a regression loss function based on the normalized Wasserstein Distance (NWD) was adopted. Experiments conducted on the Pascal VOC2007 dataset demonstrated mAP values of 40.6% and 11.4%.Zhanjun Jiang [15] proposed a small-object detection method based on APM-YOLOv7 (an improved YOLOv7 integrating ACanny, PConv-ELAN, and MGA attention). First, an adaptive Canny algorithm (ACanny) was designed to extract river contours, reducing background interference and enhancing small-object feature extraction. Second, lightweight partial convolutions (PConv) were introduced to construct an efficient layer aggregation module (PConv-ELAN), improving feature capture of morphologically diverse debris. Finally, a multi-scale gated adaptive weight allocation mechanism (MGA) was proposed to emphasize small-object features and reduce missed detections. Experiments indicated that this method improved mean average precision (mAP) by 7.02% compared with YOLOv7.Luan Qinglei et al. [16] developed PAW-YOLOv7. First, a small-object detection layer was added, and a self-attention and convolution hybrid module (ACmix) was introduced to enhance feature representation. Second, the neck network employed Omni-Dimensional Dynamic Convolution (ODConv) instead of standard convolution to reduce background interference and improve global context modeling. Third, partial convolution (PConv) was integrated into the backbone to reduce computational cost, and WIoU loss was adopted in place of CIoU to optimize regression accuracy. On the FloW-Img dataset, PAW-YOLOv7 achieved a detection accuracy of 89.7%, representing a 9.8% improvement over the original YOLOv7.Hongru Wang et al. [17] proposed an improved YOLOv8-based model. First, a reshaped network structure was constructed by integrating a high-resolution detection head and pruning redundant network layers. The backbone network from FasterNet was incorporated to optimize the C2f module, thereby reducing unnecessary computations. A fused global coordinate attention (FGCA) module was embedded in the Path Aggregation Network (PAN), and a parameter-free average attention module (PfAAM) was integrated into the Feature Pyramid Network (FPN), forming an innovative neck fusion network (GPAN + PFPN) capable of handling complex features in adverse scenarios with minimal computational burden.Lili Song et al. [18] proposed an improved YOLOv8-HSH algorithm based on YOLOv8n. The HorBlock module was enhanced to promote multi-gradient and multi-scale feature fusion, strengthening critical floating-object features. An optimized CBAM attention mechanism was applied to reduce background noise interference, significantly improving detection accuracy. Additionally, a secondary object recognition layer was added to enhance the model’s capability to detect floating objects of varying sizes across different environments. Experimental results demonstrated substantial improvements, with mAP@0.5 and mAP@0.5:0.95 increasing by 11.7% and 12.4%, respectively. Hongfeng Tao [19] proposed a novel network architecture, CDFF-YOLO, which addresses the loss of fine-grained information in small object detection by embedding a multi-branch attention module into the CDFF module, thereby enhancing the interaction between global and local features. In addition, a modified network was designed to tackle challenges such as uneven illumination and occlusion. The DFF module performs transformations and fusions of small object features extracted via spatial-to-depth convolutions in both the frequency and spatial domains, improving the network’s ability to reconstruct and integrate features across dual domains. Experimental results on the TT100K dataset demonstrate that the improved algorithm achieves gains of 3.7% and 4.8% in mAP@50 and mAP@50:95, respectively, with average precision and recall for small objects increased by 4.5% and 3.7%, while maintaining a high frame rate of 157 FPS.

Quanbo Ge et al. [20] addressed the issue of feature loss for small objects during extraction by improving the attention mechanism based on small-object features and proposing a multi-attention module, which was integrated into the feature extraction process. Additionally, an adaptive re-parameterized generalized feature pyramid network (Adaptive_RepGFPN) was introduced. On a self-constructed dataset, the proposed method achieved increases of 9.1% and 3.5% in mAP@0.5 and mAP@0.5:0.95, respectively, compared with the baseline network.References [21, 22] employed Faster R-CNN and Mask R-CNN, respectively. Compared with single-stage detectors such as YOLO, these methods exhibit higher computational complexity, resulting in relatively slower detection speeds, making them less suitable for real-time applications.

Although existing object detection algorithms have achieved significant improvements in both accuracy and inference speed, detecting small and inconspicuous floating debris on water surfaces remains a considerable challenge. The primary reasons are as follows: (1) current detection algorithms exhibit relatively low accuracy for small objects due to the lack of specialized designs targeting small targets, while datasets of water surface debris contain a high proportion of small objects, resulting in suboptimal detection performance; (2) floating debris is severely affected by noise factors such as illumination variations, water ripples, and reflections, which further reduce detection accuracy.

YOLOv8 is one of the fundamental models in the YOLO series. Compared with traditional anchor-based detection methods, its anchor-free approach offers higher detection accuracy and faster inference speed. However, in complex aquatic environments, the detection of small floating objects using YOLOv8 remains challenging due to the complex features of small targets and diverse backgrounds, leading to localization errors and insufficient target perception. To address these issues, this study proposes an improved YOLOv8-based algorithm, termed YOLOv8-SST. First, to mitigate noise interference introduced during the downsampling process of the backbone network in complex water environments, a C2fBF (C2f-BiFormer) module based on the BiFormer dual-layer routing attention mechanism was developed, which preserves fine-grained contextual feature information during feature extraction. Second, the conventional loss function was replaced with a more effective Inner-Complete Intersection over Union (Inner-CIoU) loss under auxiliary bounding boxes, enabling the model to adjust the size of auxiliary boxes more flexibly and thereby improving detection accuracy. Finally, the Adam optimizer originally used in the model was substituted with the Sophia optimizer, enhancing the model’s generalization capability.The remainder of this paper is organized as follows. Section 2 reviews the YOLOv8 model and introduces the datasets and evaluation metrics used in this study. Section 3 presents the proposed YOLOv8-SST algorithm. Section 4 reports experimental results and discussion. Finally, Section 5 outlines future research directions and concludes the study.

## Related works

### YOLOv8 network model

YOLOv8 significantly improves upon the accuracy of YOLOv5 [23] by enhancing various aspects, such as the network structure, loss functions, and sample allocation strategies. The main improvements are as follows.

**Fig 1:**
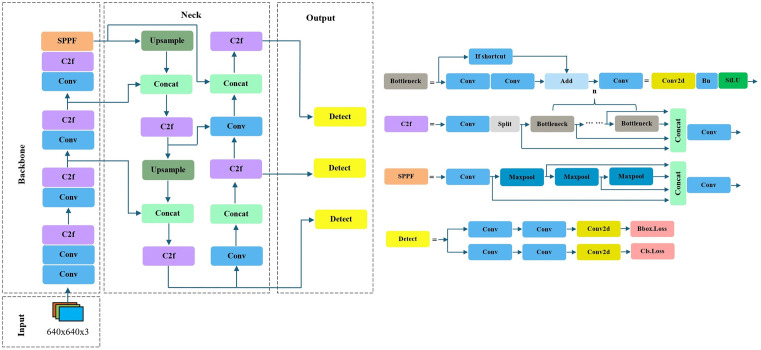
The YOLOv8 Network Model.

(1) Network Structure: The backbone of YOLOv8 retains the architecture of YOLOv5 but replaces the original C3 module with the C2f module. The design of the C2f module draws from the C3 module and the ELAN concept in YOLOv7 [24], achieving a richer gradient flow while ensuring lightweight performance. In the neck section, YOLOv8 continues to utilize the structure of YOLOv5, with the C3 module also being replaced by the C2f module. Furthermore, the convolutional module preceding the upsampling operation in the PAN-FPN is removed, allowing the feature maps derived from different stages of the backbone to be directly input into the upsampling layer. The network architecture of YOLOv8 is illustrated in Fig 1.
(2) Loss Function: The loss function of YOLOv5 comprises three components: a classification loss (cls_loss), a regression loss (box_loss), and a confidence loss (obj_loss). In contrast, YOLOv8 employs only the varifocal loss [25] for classification. The primary advancement of the varifocal loss is the introduction of asymmetric weighting, which effectively addresses the issue of imbalanced numbers of positive and negative samples.
(3) Sample Allocation Strategy: YOLOv5 uses a static allocation strategy. Considering the advantages of dynamic allocation strategies, YOLOv8 adopts the task-aligned assigner [26] for matching positive and negative samples, implementing an anchor-free method instead of the IoU-based matching or single-sided ratio allocation schemes of anchor-based methods. The matching strategy of the task-aligned assignment approach selects positive samples based on scores weighted by classification and regression metrics.

### Dataset construction

The dataset used in this study originates primarily from the inland floating debris dataset (FloW) [27], the water surface object detection dataset (WSODD) [28], and commonly encountered water surface floating objects collected independently.

The FloW dataset is the first floating debris detection dataset developed from the perspective of unmanned surface vehicles, drawing attention to the cleanup of floating debris in inland waters while also serving as a reference for research on the detection of small targets on the surface of water. Its subset, FloW-Img, contains 2,000 images and 5,271 labeled objects with varying sizes. Additionally, the FloW dataset provides 200 unlabeled video sequences of water surfaces, offering further insights for target detection algorithm research. The WSODD, developed by Zhou et al., is a large-scale, high-quality benchmark dataset proposed for autonomous water surface driving; it comprises 7,467 water surface images and contains 14 categories and 21,911 labeled instances. In this study, 564 images are manually selected from the WSODD and incorporated into the FloW-Img subset.

Given that the types of floating debris contained in the FloW dataset and the WSODD are relatively uniform, we independently capture 2,400 images of floating debris, resulting in a total of 4,964 images of floating debris on the surface of water. The dataset includes 1,600 images of normal water surfaces and 3,364 images of floating debris, which are categorized as follows: 1,950 images of bottle-type debris, 172 images of grass-type debris, 395 images of branch-type debris, 190 images of milk box-type debris, 155 images of plastic bag-type debris, 252 images of leaf-type debris, 100 images of ball-type debris, and 150 images of plastic garbage-type debris. Considering the significant proportion of bottle-type debris and the relatively small proportions of other types, the dataset is classified into two categories: Bottle and Garbage. Utilizing LabelImg, all 4,964 images are reannotated in the YOLO format and categorized into two classes: Garbage and Bottle. To satisfy the experimental requirements, the dataset is divided into training, validation, and test sets at a ratio of 6:2:2.

### Evaluation metrics

This study evaluates the performance of the tested models via the mAP, model computational cost (giga floating-point operations per second (GFLOPs)), and number of frames per second (FPS). The mAP is utilized to assess the accuracy of the model, and its calculation formula is as follows:


$$\begin{array}{c} mAp=\sum \frac{P_{A}}{N} \end{array}$$ (1)

where $\;P_{A}$ represents the area under the curve formed by precision on the x-axis and recall on the y-axis and N denotes the total number of detection classes. mAP@0.5 indicates the average precision (AP) achieved for each class calculated at an IoU threshold of 0.5, followed by averaging the AP values across all classes. mAP@0.5:0.95 refers to the computation of the mAP for IoU thresholds ranging from 0.5 to 0.95 in increments of 0.05, with the final mAP being the average of these values.

## Methodology

In this study, YOLOv8n was selected as the base model due to its low parameter count and fast detection speed, making it suitable for the proposed algorithm. Building upon the YOLOv8 framework, the YOLOv8-SST algorithm is proposed. Compared with the original YOLOv8, the YOLOv8-SST algorithm introduces the following improvements: (1) a C2fBF (C2f-BiFormer) module based on the BiFormer dual-layer routing attention mechanism, which preserves fine-grained contextual feature information during feature extraction; (2) a more effective Inner-Complete Intersection over Union (Inner-CIoU) loss under auxiliary bounding boxes, replacing the original loss function; and (3) substitution of the Adam optimizer with the Sophia optimizer, enhancing the model’s generalization capability.

### Improvements made to the backbone network

**Fig 2:**
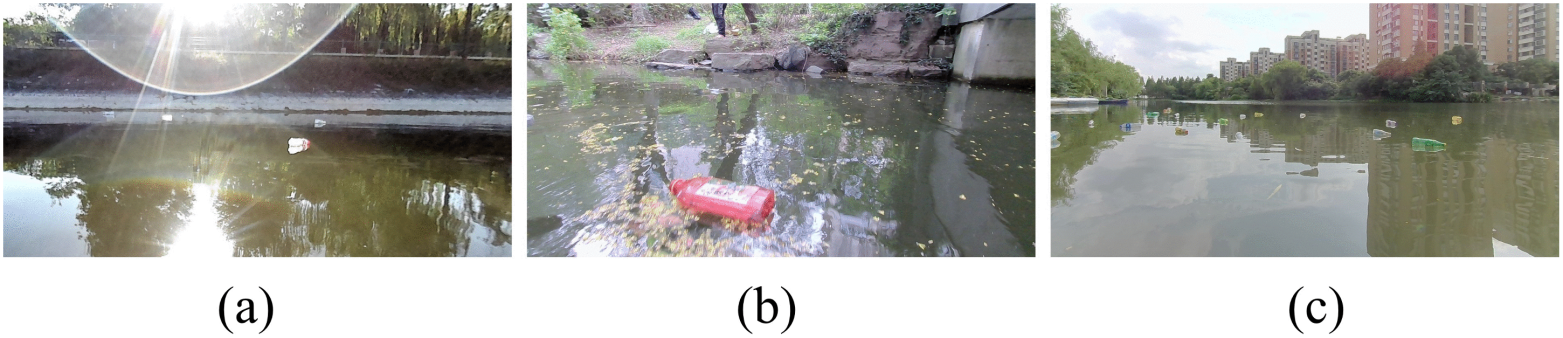
Water surface interference factors.

In complex aquatic environments, images are easily affected by various interference factors such as weather conditions, illumination changes, and water surface reflections, resulting in multiple types of noise. Specifically, strong lighting can cause intense reflections on the water surface, as shown in Fig 2(a); insufficient ambient lighting during image capture can lead to blurring and distortion, as illustrated in Fig 2(b); and the presence of densely distributed floating objects can cause overlap between objects, as depicted in Fig 2(c).

During the process of feature extraction from such scene images using convolutional neural networks (CNNs), substantial noise is often introduced. This noise disrupts the long-range dependencies among pixels in the feature maps, thereby weakening the model’s capability to detect and recognize water-surface targets, which may ultimately result in false detections or missed detections.

To mitigate the adverse impact of noise on model performance, this study incorporates a mechanism capable of capturing long-range dependencies to enhance target detection and recognition in water-surface scenarios. BiFormer, as a variant of the Transformer, employs a bi-level routing attention mechanism that enables more flexible computational allocation and content-aware processing. This design allows dynamic attention computation, thereby facilitating more accurate extraction of critical features.


$$\begin{array}{c} A^{r}=Q^{r}{\left(K^{r}\right)}^{T} \end{array}$$ (2)

$$\begin{array}{c} I^{r}=topkIndex\left(A^{r}\right) \end{array}$$ (3)

$$\begin{array}{c} K^{g}=gather\left(K,I^{r}\right),V^{g}=gather\left(V,I^{r}\right) \end{array}$$ (4)

$$\begin{array}{c} O=Attention\left(Q,K^{g},V^{g}\right)+LCE(V) \end{array}$$ (5)

**Fig 3:**
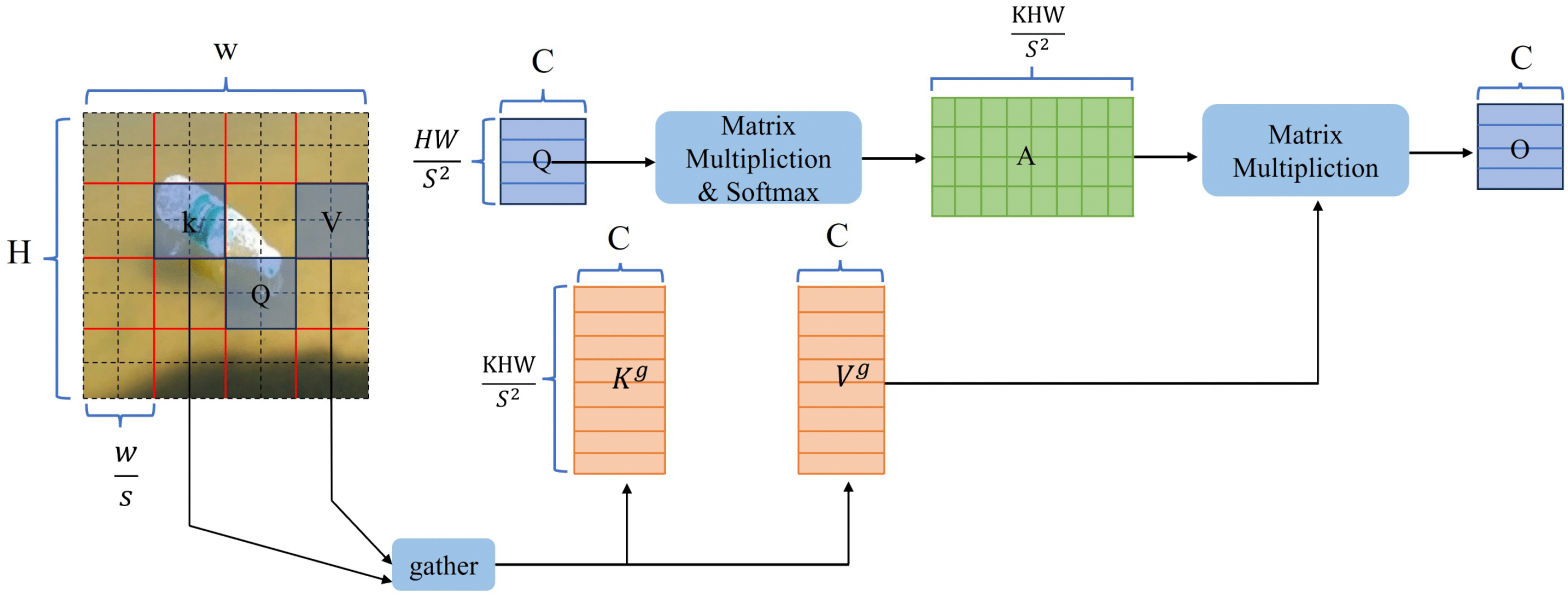
Bi-level attention mechanism.

As illustrated in Fig 3, the bi-level routing attention mechanism first divides the input feature map $\text{X}\in R^{H\times W\times C}$ into S*S non-overlapping regions, thereby transforming X into $X^{r}\in R^{S^{\text{2}}\times HWC/S^{\text{2}}}$. A fully connected layer is then applied to linearly project $X^{r}$, generating Q,K and V. The mean values of Q and K are subsequently computed to obtain $Q^{r}$, $K^{r}$ and $Q^{r}$, $K^{r}$, respectively. The adjacency matrix $A^{r}$, which measures semantic similarity between different regions, is calculated according to Eq. (2). Next, Eq. (3) filters the matrix $A^{r}$ to select the top-*k* regions with the highest semantic similarity, resulting in $I^{r}$, where the i-th row of $I^{r}$ contains the indices of the k regions most relevant to the ii-th region. Using $I^{r}$, Eq. (4) filters and gathers k and V to produce $K^{g}$ and $V^{g}$. Finally, fine-grained token-to-token attention is applied to Q, $K^{g}$, and $V^{g}$, as expressed in Eq. (5), where LCE denotes a depthwise separable convolution with a kernel size of 5 and a stride of 1.

**Fig 4:**
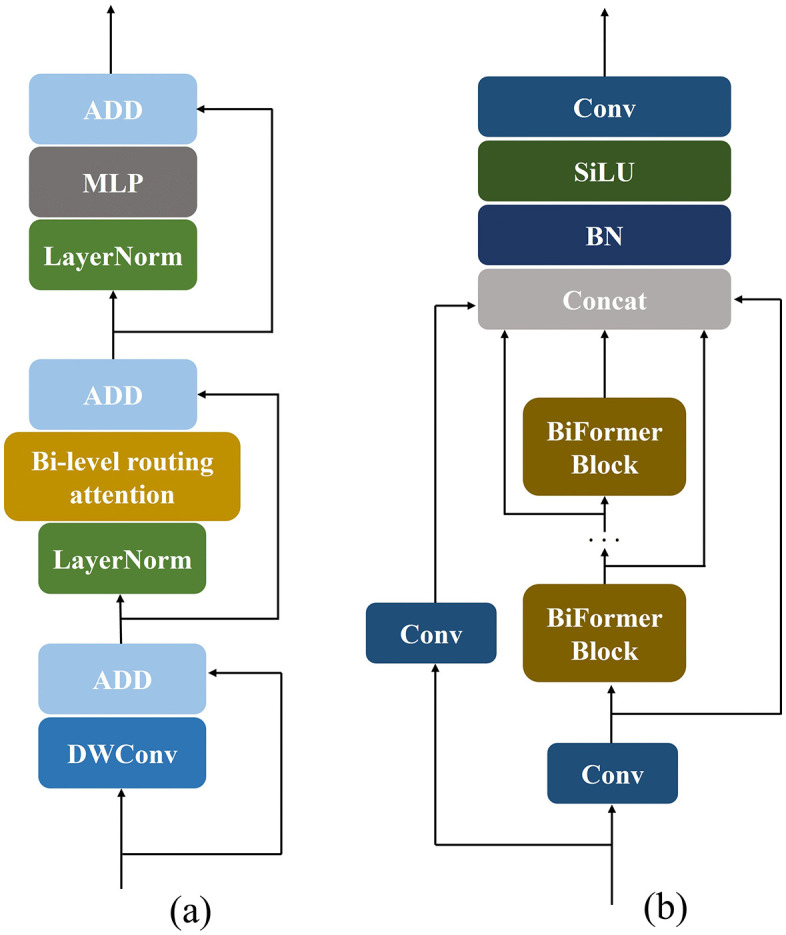
BiFormerBlock, C2fBF.

To mitigate noise interference and improve small object detection on water surfaces, we introduce the C2fBF (C2f-BiFormer) module, which integrates the BiFormerBlock with the C2f structure. Specifically, the bi-level routing attention mechanism filters out most irrelevant key–value pairs at the coarse region level, thereby reducing redundant information and retaining only the most relevant routing regions for fine-grained attention computation. Compared with global self-attention, this design significantly decreases computational cost and memory consumption while enhancing the capture of critical dependencies.As illustrated in Fig 4(a), the BiFormerBlock first employs a depthwise separable convolution (DWConv) to implicitly encode relative positional information, followed by bi-level routing attention and a multilayer perceptron (MLP) to perform cross-position modeling and position-wise embedding. In parallel, the C2f structure introduces multiple cross-layer branch connections, enriching gradient flow and facilitating more effective learning of residual features.Building upon these advantages, the proposed C2fBF module replaces one C2f block in the backbone network (Fig 4(b)). This design preserves the lightweight nature of the model while enabling fine-grained contextual feature retention and robust residual learning, thereby reducing noise interference and improving the modeling of long-range dependencies in small water-surface object detection.

The proposed C2fBF module combines the cross-stage C2f connections with the BiFormerBlock, which employs a bi-level routing attention mechanism to capture long-range dependencies while reducing noise interference in water surface images. Let the input feature map be $X\in R^{H\times W\times C}$. First, X is partitioned into S×Snon-overlapping regions, producing a reshaped representation $X^{r}\in R^{S^{\text{2}}\times \frac{HWC}{S^{\text{2}}}}$. Linear projections are applied to obtain queries $Q=X^{r}W_{Q}$, keys $K=X^{r}W_{K}$, and values $V=X^{r}W_{V}$, where $W_{Q},W_{K},W_{V}\in R^{\frac{HWC}{S^{\text{2}}}\times d}$ and d is the latent embedding dimension. The adjacency matrix is computed as follows:


$$\begin{array}{c} A^{r}=Q^{r}{\left(K^{r}\right)}^{T}\in R^{S^{\text{2}}\times S^{\text{2}}} \end{array}$$ (6)

where $Q^{r}$ and $K^{r}$ denote the mean-pooled queries and keys for each region, capturing semantic similarity between regions. To reduce computational overhead, only the topk regions per row with the highest semantic similarity are retained:


$$\begin{array}{c} I_{i}^{r}=\text{t}opk\left(A_{i}^{T}\right),{\;\;k}^{g}=\text{g}ather\left(K,I\left(K,I^{T}\right)\right),\;\;{\;\;V}^{g}=\text{g}ather\left(V,I\left(V,I^{T}\right)\right) \end{array}$$ (7)

where $I_{i}^{r}$ contains indices of the top-k regions most relevant to the i-th region. The final attention output is computed as follows:


$$\begin{array}{c} O=\text{A}ttention\left(Q,k^{g},V^{g}\right)+LCE(V) \end{array}$$ (8)

where LCE denotes a depthwise separable convolution with kernel size 5 and stride 1. The FLOPs for adjacency matrix computation are $O(S^{\text{4}}\cdot d)$, and the sparse attention over top-k regions reduces the subsequent attention complexity to $O(S^{\text{2}}\cdot k\cdot d)$, with $k\ll S^{\text{2}}$. The LCE adds O(HWC)FLOPs, which is negligible compared to standard convolution.

By replacing a single C2f module in YOLOv8n with C2fBF, the parameter count increases moderately by $\Delta P\approx \text{3}d^{\text{2}}+d\cdot d_{\text{MLP}}$, while FLOPs increase by $\Delta F\approx S^{\text{2}}\cdot k\cdot d+HWC$. Empirically, YOLOv8-SST maintains near real-time inference speed, indicating that the improved detection accuracy and preserved fine-grained contextual features are achieved with a moderate computational overhead, suitable for small object detection in complex aquatic environments.

### Improvement provided for the loss function

The Inner-CIoU loss function, which integrates the concepts of Inner-IoU [29] and Complete IoU (CIoU) [30], is designed to enhance the detection performance for small objects as well as the overall detection accuracy. Specifically, the Inner-IoU loss introduces a penalty term tailored for small objects on the basis of the conventional IoU. This loss function exhibits multiple advantages, particularly when handling small objects or partially overlapping bounding boxes, where its sensitivity and robustness are more prominent. Unlike the traditional IoU, which may yield excessively high loss values during optimization when bounding boxes only partially overlap, Inner-IoU focuses on the overlapping area within the bounding boxes. This design ensures that even when small errors exist between bounding boxes, the loss function remains highly sensitive. In addition, Inner-IoU leverages a scaling factor, termed *ratio*, to generate auxiliary bounding boxes of varying scales for loss computation. By tuning the *ratio* parameter, the penalty term can be adapted to the requirements of different detection tasks.

Complete IoU (CIoU) is an extension of the conventional IoU loss function, incorporating additional geometric factors to achieve more precise geometric alignment between the predicted and ground-truth bounding boxes while accelerating the convergence process. Specifically, CIoU introduces a penalty term based on the distance between the centers of the two boxes, thereby ensuring that the predicted bounding box is more accurately aligned with the ground truth in terms of spatial location.

Therefore, the Inner-CIoU loss function integrates the improvements of both loss functions, which not only enhances the detection performance for large objects but also significantly optimizes the detection of small objects, while simultaneously accelerating the model convergence to a certain extent. The formulation of this loss function is expressed as follows:


$$\begin{array}{c} Loss_{\text{Inner}-\text{CIoU}}=\text{1}-IoU+\frac{\rho^{\text{2}}\left(b,b_{\text{gt}}\right)}{c^{\text{2}}}+a\cdot V+\lambda \cdot Penalty \end{array}$$ (9)

Among them, a·V primarily serves to reduce the aspect ratio discrepancy between the predicted and ground-truth bounding boxes, thereby ensuring closer shape alignment between the two.

### Sophia optimizer

The Adam algorithm [31] is an optimization method based on adaptive moment estimation. It calculates first-order and second-order moment estimates of the model gradients, designing independent adaptive learning rates for different model parameters. This allows each parameter to retain its own learning rate, thereby enhancing the performance of the model parameters [32]. However, the Adam optimizer tends to converge to sharp minima with large curvatures, which may lead to poor generalization performance. Therefore, while Adam has a fast convergence speed, its generalization performance is suboptimal. This paper introduces a new optimizer, Sophia [33], to accelerate the convergence speed and accuracy of the model.

Sophia is a lightweight second-order optimizer that uses an inexpensive random estimate of the Hessian diagonal as a preprocessor and controls the worst-case update size through a clipping mechanism. The Hessian is the matrix of second-order partial derivatives, which describes the local curvature of a function at a given point. For a function, the elements of the corresponding Hessian matrix H are given by Eq. (7):


$$\begin{array}{c} H_{ij}=\frac{\partial^{\text{2}}f}{\partial x_{i}\partial x_{j}} \end{array}$$ (10)

In the above equation, H is a symmetric matrix that reflects the second-order derivative information of the given function.

Sophia estimates the Hessian diagonal only over a few iterations, allowing the average time and memory cost incurred per step to be negligible. Inspired by the exponential moving average (EMA) operation applied to gradient moments in the Adam optimizer, Sophia employs a noise-reduction technique by updating the EMA every k steps across the iterative process, thereby providing a new rule for Hessian estimation diagonal, as shown in Eq. (8):


$$\begin{array}{c} h_{t}=\beta_{\text{2}}h_{t-k}+\left(\text{1}-\beta_{\text{2}}\right){\hat{h}}_{t}\;\text{if}(t\;\text{mod}\;k=\text{1});\text{else}(h_{t}=h_{t-\text{1}}) \end{array}$$ (11)

In this equation, $h_{t}$ represents the state at time step  t, $h_{t-k}$ denotes the state at time step t−k, and ${\hat{h}}_{t}$ is the new candidate state computed at time step t. The parameter $\beta_{\text{2}}$ is a weighting parameter (ranging between 0 and 1) that is used to smooth the transition between the new and old states, and  t mod k calculates the remainder of t  divided by  k.

On nonconvex functions, using the Hessian as a preconditioner may lead to convergence at local maxima instead of local minima. Furthermore, Hessian estimation inaccuracies and changes in the Hessian along the trajectory can make the obtained second-order information unreliable. To address this issue, we can (1) consider only the positive elements of the Hessian diagonal and (2) introduce coordinate clipping for the updates, where all operations are applied in a coordinatewise manner. The updated formulation is given in Eq. (9):


$$\begin{array}{c} \theta_{t+\text{1}}\leftarrow \theta_{t}-\eta_{t}\cdot \text{clip}\left(\frac{m_{t}}{\text{max}\left\{\gamma \cdot h_{t},\in \right\}},\text{1}\right) \end{array}$$ (12)

In the above equation, $\theta_{t}$ represents the parameter vector at iteration t, $\eta_{t}$ denotes the learning rate at iteration t, $m_{t}$ indicates the gradient moving average at iteration t, $h_{t}$ is the Hessian diagonal estimate at the t, γ is a scaling factor used to adjust the ${\;h}_{t}$ value, and ∈>0 is a very small constant that prevents division by zero.

**Fig 5:**
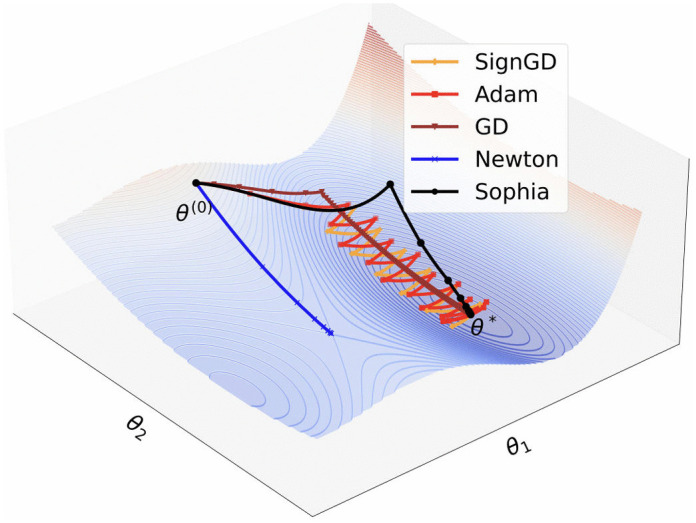
Comparison of trajectories of various optimization algorithms in a two-dimensional loss function space.

Fig 5 illustrates the behaviors of several optimization algorithms on a two-dimensional loss function, including signSGD [34], Adam, Gradient Descent (GD), Newton’s Gradient Descent, and Sophia. The signSGD trajectory exhibits noticeable oscillations, indicating considerable fluctuations during the search for the optimal solution and a relatively slow convergence speed. Although Adam demonstrates a smoother trajectory and faster convergence, its excessive smoothness may cause the algorithm to become trapped in a local optimum rather than reaching the global minimum. The GD algorithm converges very slowly, while the Newton method tends to converge to a saddle point. In contrast, Sophia advances rapidly along both dimensions and is able to converge to the minimum within just a few steps.

By updating the model optimizer, the floating debris detection model can adapt better to complex water surface environments, thereby improving the efficiency of the floating debris detection process. The improved optimizer significantly reduces the confidence loss induced when complex targets that are easily hidden are detected. The updates made by the Sophia optimizer result in more stable precision curves for various detection targets, making it more suitable for detecting complex objects on water surfaces.

**Fig 6:**
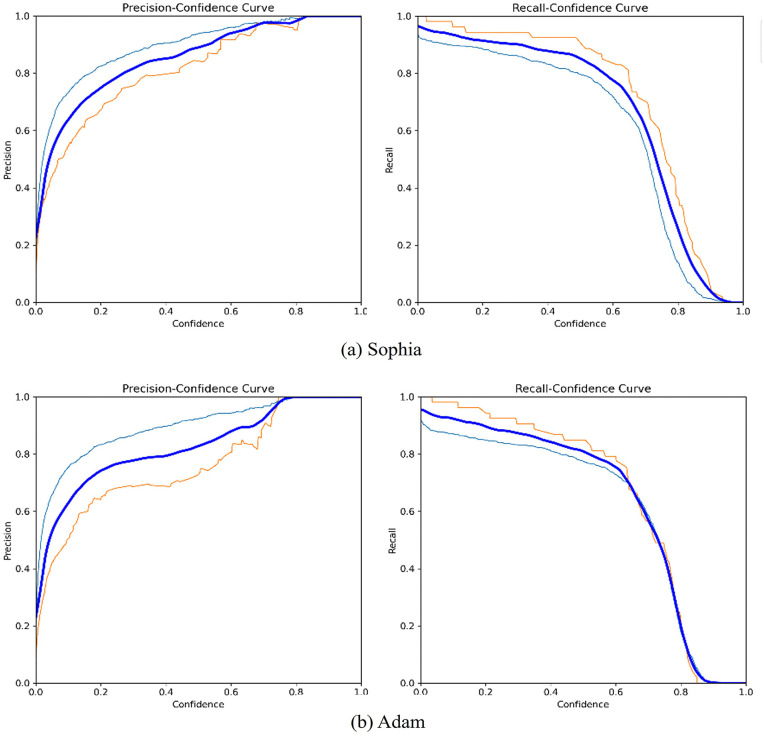
Comparison of Sophia and Adam in terms of mAP.

By updating the model optimizer, the floating object detection model can better adapt to complex water-surface environments, thereby improving the efficiency of water-surface object detection. The experimental evaluation employed Precision–Confidence and Recall–Confidence curves as metrics. A comparison of experimental results before and after the optimizer update is shown in Fig 6. By examining the two sets of curves, it can be observed that the optimizer improvement substantially reduces confidence loss when detecting challenging and easily occluded targets. The updates introduced by the Sophia optimizer result in more stable precision curves across various detection targets, making it better suited for complex water-surface detection scenarios.

## Experimental results and analysis

### Comparison among the effects of the improvement methods

To explore the practical effects of the proposed improved algorithm, we conducted experiments to analyze the impacts of the weighted loss function and optimizer on the resulting model performance.

By comparing the Inner-CIoU in Eq. (6) with DIoU [30], GIoU [35], EIoU [36], and CIoU, it can be seen from Table 1 that YOLOv8 using the Inner-CIoU loss function achieves the best performance in terms of mAP. Compared with the original CIoU-based algorithm, it improves mAP@0.5 by 0.6% and mAP@0.5:0.95 by 0.3%.

**Table 1:** Comparison among the tested models after applying the improved loss function.

Model	mAP@0.5 (%)	mAP@0.5:0.95 (%)	GFLOPs	FPS (f/s)
YOLOv8n + IoU	85.2	53.9	8.2	108
YOLOv8n + DIoU	86.1	54.1	8.2	109
YOLOv8n + GIoU	86.4	54.6	8.2	108
YOLOv8n + EIoU	87.9	55.0	8.2	107
YOLOv8n + CIoU	88.1	55.3	8.2	108
YOLOv8n + Inner-CIoU	**88.7**	**55.6**	**8.2**	**108**

Since the improvements provided for the optimizer did not affect the GFLOPs and FPS parameters, the proposed Sophia optimizer significantly outperformed the original Adam optimizer, with increases of 1.1% and 0.5% in terms of the mAP@0.5 and mAP@0.5:0.95 metrics. Compared with other commonly used optimizers, such as weighted Adam (AdamW) [37], RAdam [38], Nadam [39], Adamax [31], and root-mean-square propagation (RMSProp), Sophia had the best performance. The specific results are shown in Table 2.

**Table 2:** Comparison among the tested models after applying the improved optimizer.

Model	mAP@0.5 (%)	mAP@0.5:0.95 (%)
YOLOv8n + RMSProp	79.8	47.6
YOLOv8n + Adamax	83.7	52.0
YOLOv8n + NAdam	86.1	54.8
YOLOv8n + RAdam	88.1	56.7
YOLOv8n + Adam	88.1	55.3
YOLOv8n + AdamW	88.8	56.9
YOLOv8n +Sophia	**89.3**	**57.2**

### Ablation experiment

**Fig 7:**
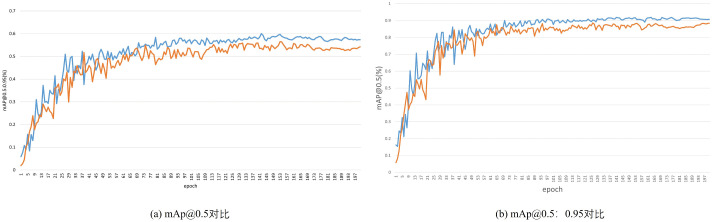
Comparison between the mAPs of YOLOv8-SST and YOLOv8n.

As shown in Table 3, the introduction of the SPPF-LSKA module resulted in increased detection accuracy, with mAP@0.5 and mAP@0.5:0.95 improving by 1.0% and 1.3%, respectively. The incorporation of the NWD regression loss function also enhanced the detection performance achieved by the model for small targets, with increases of 0.6% in mAP@0.5 and 1.1% in mAP@0.5:0.95. When both the SPPF-LSKA module and the NWD regression loss function were used together, the improvements were 1.3% and 4.3% in terms of mAP@0.5 and mAP@0.5:0.95, respectively. Finally, replacing the Adam optimizer with Sophia significantly increased the overall model performance, yielding 1.1% and 1.8% increases in both mAP@0.5 and mAP@0.5:0.95. Fig 7 shows the changes between the mAPs of the improved model and the original model. Although the detection speed (FPS) decreased, it remained at a high rate of 86 f/s.

**Table 3:** Results of the ablation experiment.

C2fBF	Inner-CIoU	Sophia	mAP@0.5 (%)	mAP@0.5:0.95 (%)	GFLOPs	FPS (f/s)
—	—	—	88.1	55.3	8.2	108
✔	—	—	89.1	56.6	12.0	92
—	✔	—	88.7	56.4	8.2	108
—	—	✔	89.2	57.1	8.2	108
✔	✔	—	89.4	59.7	12.9	90
✔	✔	✔	**91.2**	**60.3**	**12.9**	**86**

### Comparison among different models in detection experiments

To validate the superiority of the proposed YOLOv8-SST algorithm in terms of detecting floating debris on water surfaces, we conducted comparisons with mainstream object detection algorithms (including Faster R-CNN; SSD; the YOLO series, namely, YOLOv5, YOLOv7, and Gold-YOLO-S [39]; and the YOLOv8 model incorporating a generalized feature pyramid network (GFPN) [40] and the YOLOv8 model using partial convolution (Pconv) [41] using the same dataset and experimental conditions. All experiments were performed on a workstation equipped with an NVIDIA RTX 4090 GPU (24 GB VRAM), an Intel Core i9-13900K CPU, and 64 GB of RAM. The proposed YOLOv8-SST model was implemented in PyTorch 2.0.1 and accelerated with CUDA 11.8. Input images were resized to 640 × 640 pixels during training. The initial learning rate was set to 0.001, with a batch size of 16, and the Sophia optimizer was used, configured with a momentum of 0.937 and a weight decay of 0.0005. To ensure reproducibility and fair comparison, all baseline models compared in this study were trained under identical conditions, including hyperparameter settings.

Table 4 shows that the proposed algorithm improved the mAP@0.5 values by 5.0%, 2.7%, 3.1%, and 3.7%. Similarly, the mAP@0.5:0.95 values increased by 7.6%, 4.6%, 5.0%, and 7.2%. Compared with YOLOv5s and YOLOv7-tiny, the proposed algorithm achieved detection speed enhancements while reducing the imposed computational complexity, with improvements of 3 f/s and 14 f/s, respectively. Although the detection speed of the proposed algorithm was lower than that of YOLOv8n, it still met practical detection requirements. Additionally, the proposed algorithm demonstrated better computational efficiency than Gold-YOLO-S did.

**Table 4:** Performance comparison among different models.

Model	Input size	mAP@0.5 (%)	mAP@0.5:0.95 (%)	GFLOPs	FPS (f/s)
SSD	512 × 512	55.6	38.2	273.7	47
Faster R-CNN	720 × 720	71.5	40.7	947.3	22
YOLOv5s	640 × 640	86.2	52.7	15.8	83
Gold-YOLO-S	640 × 640	87.5	53.1	46.0	90
YOLOv8n	640 × 640	88.1	55.3	8.2	108
YOLOv7-tiny	640 × 640	88.5	55.7	13.0	72
YOLOv8-PConv	640 × 640	88.7	57.3	4.14	46
YOLOv8-GFPN	640 × 640	89.1	58.9	4.07	78
YOLOv8-SST	640 × 640	**91.2**	**60.3**	**12.9**	**86**

YOLOv8-PConv is particularly effective at handling missing or incomplete input information, as it adaptively ignores invalid areas to avoid erroneous feature extraction results. Moreover, YOLOv8-GFPN effectively integrates features from different scales, which is crucial for detecting multiscale targets and can provide increased detection accuracy. However, the lightweight nature of both models does not provide a significant advantage in real-time detection cases.

In conclusion, the improved YOLOv8-SST algorithm substantially reduced the imposed computational complexity while achieving significant detection speed and average precision gains over the Faster R-CNN and SSD networks. Thus, the modified YOLOv8-SST algorithm not only significantly improved the detection accuracy but also maintained a high detection rate, outperforming the current mainstream detection algorithms and other modified algorithms.

**Fig 8:**
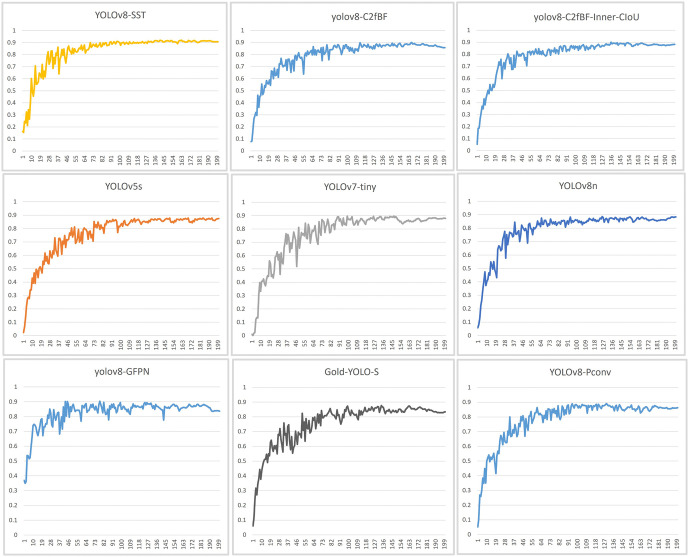
mAP@0.5 Changes Relative to the YOLO Series of Algorithms.

Fig 8 shows the mAP changes observed with respect to various YOLO series algorithms, including YOLOv5s, Gold-YOLO-S, YOLOv7-tiny, YOLOv8n, YOLOv8-PConv, YOLOv8-GFPN, and the proposed YOLOv8-SST algorithm. After 200 iterations of training, YOLOv8-SST achieved the best mAP performance and was the only algorithm that remained stable above 90%.

### Visual analysis

**Fig 9:**
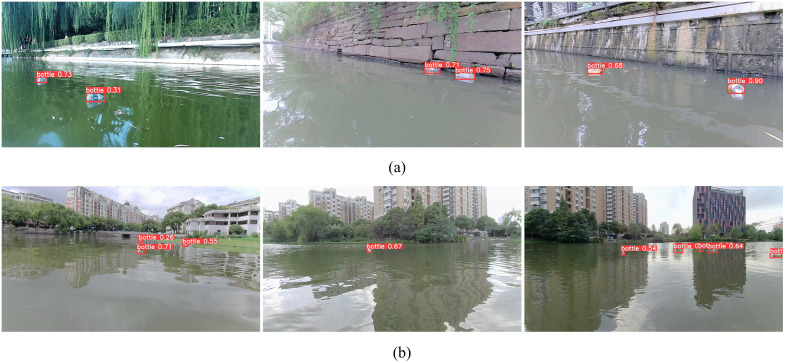
Visualization of water-surface object detection for different object sizes.

**Fig 10:**
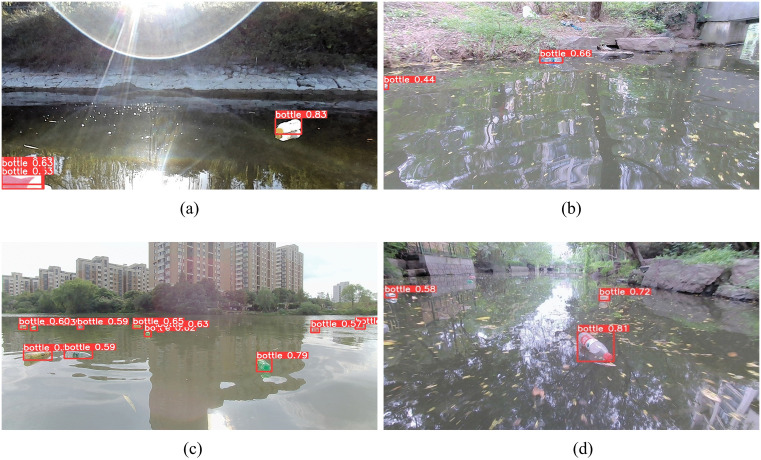
Visualization of water-surface object detection under different backgrounds.

To validate the effectiveness of the proposed improved algorithm for detecting floating objects on actual water surfaces, floating objects were selected from the experimental test set for evaluation purposes. This included floating objects with various sizes, those in different backgrounds, and those under different lighting conditions, with the detection results shown in Figs 9 and 10. Fig 9(a) shows that the proposed algorithm performed well in terms of detecting large targets. Fig 9(b) shows that the algorithm accurately detected a significant number of small targets, with no obvious false positives or missed detections, indicating good robustness and generalizability; thus, our method effectively meets the detection needs of real-world scenarios.

To further evaluate the detection performance of the proposed algorithm, YOLOv8-SST was tested under various challenging conditions: strong lighting backgrounds (Fig 10(a)), complex water-surface backgrounds (Fig 10(b)), dense object backgrounds (Fig 10(c)), and blurred or distorted backgrounds (Fig 10(d)). The YOLOv8-SST algorithm is minimally affected by changes in illumination and demonstrates strong robustness against environmental noise. It accurately detects targets on complex water surfaces, and detection performance remains reliable even under blurring or occlusion, substantially mitigating the issue of missed detections.

**Fig 11:**
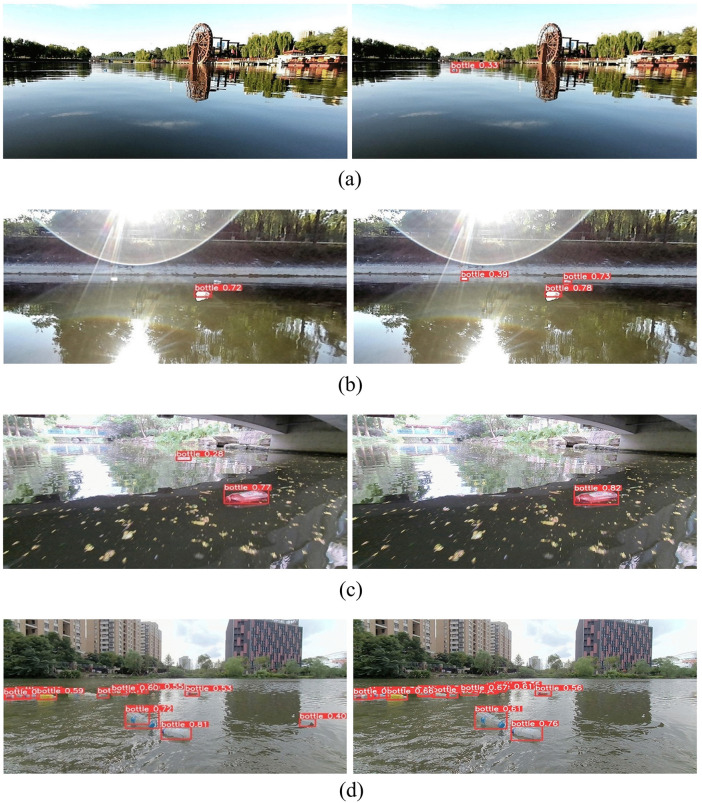
Visualization comparison between YOLOv8n and YOLOv8-SST.

Fig 11 compares the results of the baseline model YOLOv8n with the proposed YOLOv8-SST algorithm. In each sub-figure, the left image shows the detection results from the YOLOv8n algorithm, while the right image displays the results from the YOLOv8-SST algorithm. Fig 11(a) compares small object detection, revealing that the improved algorithm excels in detecting small targets. Fig 11(b) highlights that under strong lighting, the original algorithm suffers from significant missed detections. In Fig 11(c), in a complex distorted background, the original algorithm incorrectly identifies the specular highlights on the water surface as target objects. Fig 11(d) shows that in a dense background, the original algorithm fails to detect all target objects completely. Overall, the improved algorithm outperforms the baseline algorithm in detecting small targets and under various backgrounds.

**Fig 12:**
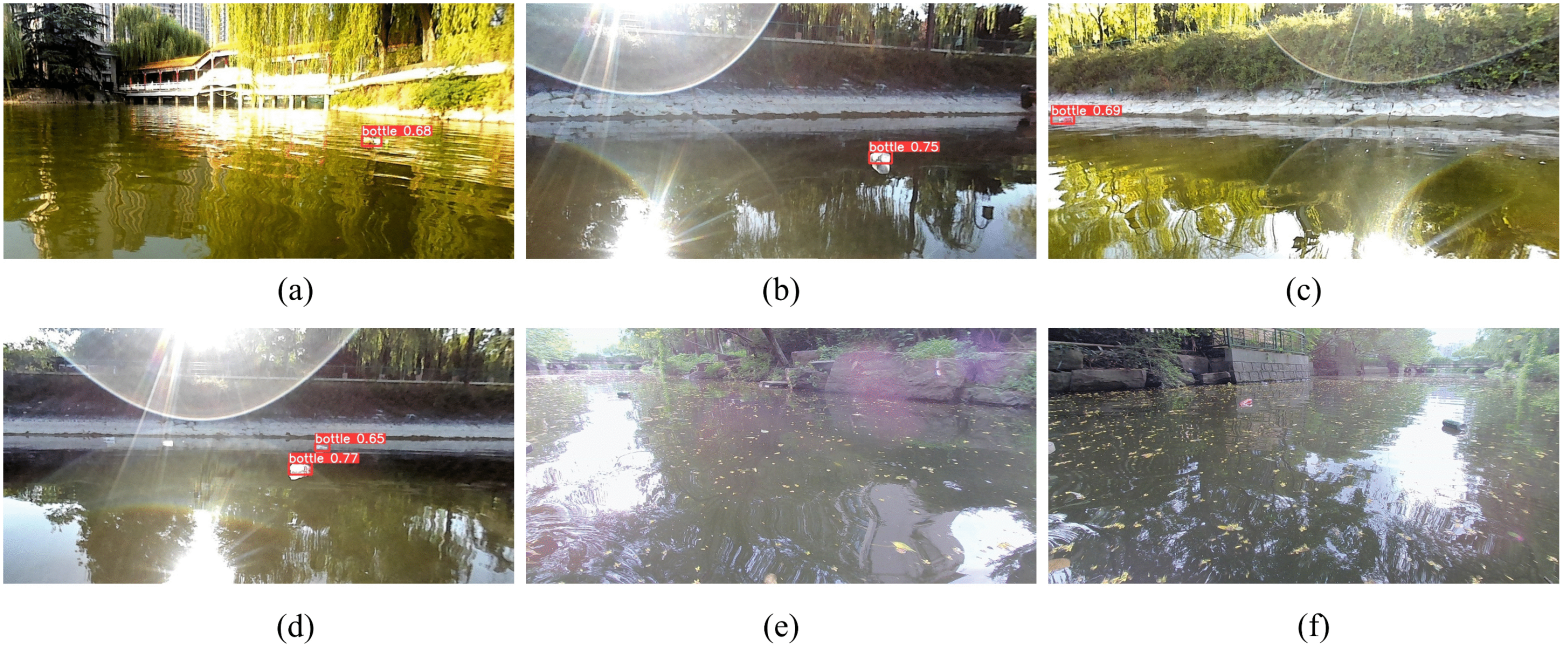
Model performance under diverse illumination environments.

Fig 12 illustrates the detection performance of the model under six different illumination conditions. The model successfully identifies floating objects in Fig 12(a), 12(b), and 12(c). However, Fig 12(d), 12(e), and 12(f) exhibit varying degrees of missed detections, including complete failures to detect targets. Although the model achieves a high overall mAP score, its robustness in complex aquatic environments remains insufficient. This limitation is primarily attributed to the unique optical properties of water surfaces—such as strong reflections, high-glare regions, reduced visibility—as well as the dynamic behavior of floating objects affected by wave-induced motions.These failure cases highlight inherent weaknesses of the current model and provide valuable insights for future improvements. Specifically, they underscore the necessity of developing more illumination-invariant and reflection-resistant network architectures, along with more comprehensive and realistic data augmentation strategies that capture extreme lighting variations in aquatic environments.

## Conclusion

This study proposes an improved YOLOv8n-based algorithm, YOLOv8-SST, for detecting floating objects on water surfaces, with a focus on mitigating noise interference caused by complex water environments and significantly enhancing the detection performance and accuracy for small objects. The main improvements are as follows: First, a C2fBF (C2f-BiFormer) module, constructed based on the BiFormer bi-level routing attention mechanism, is introduced to preserve fine-grained contextual information during feature extraction, effectively reducing noise generated during the sampling process in complex water scenarios. Second, the conventional loss function is replaced with a more effective Inner-Complete Intersection over Union (Inner-CIoU) loss, which allows the model to flexibly adjust auxiliary bounding boxes during detection, thereby improving detection accuracy. Third, the Sophia optimizer is employed instead of Adam, further enhancing the model’s generalization and adaptability across diverse scenarios.

Compared with the original YOLOv8n algorithm, YOLOv8-SST exhibits increased computational complexity; future work will focus on optimizing the network structure to improve efficiency. Additionally, expanding the input dataset—particularly for seven categories of floating objects including aquatic plants, branches, milk cartons, plastic bags, plastic debris, balls, and leaves—can further enhance the algorithm’s performance for multi-class floating object detection tasks.

In addition to the technical contributions, the proposed floating-object detection algorithm has direct relevance to water pollution monitoring. By accurately identifying debris and other floating pollutants, the model can support practical environmental applications such as autonomous cleanup by unmanned surface vehicles, early-warning systems for pollution accumulation, and large-scale water quality surveillance. These capabilities can improve the efficiency and timeliness of pollution management in rivers, lakes, and coastal areas. At the same time, practical deployment faces challenges, including sensitivity to extreme lighting, strong reflections, and highly dynamic water surfaces. Future work will focus on enhancing robustness under these conditions, expanding dataset diversity to cover more real-world scenarios, and integrating temporal information to ensure consistent performance in complex aquatic environments. Furthermore, the proposed YOLOv8-SST algorithm can be readily extended to other tasks involving small object detection in natural environments. Its robust feature extraction and attention mechanisms enable accurate detection of small and visually challenging targets, making it suitable for applications such as wildlife monitoring, aerial surveillance, and environmental observation, where small-scale objects often appear under complex and dynamic conditions.
